# Defined tetra-allelic gene disruption of the 4-coumarate:coenzyme A ligase 1 (Pv4CL1) gene by CRISPR/Cas9 in switchgrass results in lignin reduction and improved sugar release

**DOI:** 10.1186/s13068-017-0972-0

**Published:** 2017-11-30

**Authors:** Jong-Jin Park, Chang Geun Yoo, Amy Flanagan, Yunqiao Pu, Smriti Debnath, Yaxin Ge, Arthur J. Ragauskas, Zeng-Yu Wang

**Affiliations:** 1 Noble Research Institute, Ardmore, OK 73401 USA; 2 BioEnergy Science Center, Oak Ridge National Laboratory, Oak Ridge, TN 37831 USA; 3 Joint Institute for Biological Sciences, Biosciences Division, Oak Ridge National Laboratory, Oak Ridge, TN 37830 USA; 4 Department of Chemical and Biomolecular Engineering, University of Tennessee, Knoxville, TN 37996 USA; 5 Center for Renewable Carbon, Department of Forestry, Wildlife, and Fisheries, University of Tennessee Institute of Agriculture, Knoxville, TN 37996 USA

**Keywords:** Genome editing, CRISPR/Cas9, Switchgrass, *Panicum virgatum*, Bioenergy, Lignin biosynthesis, 4-Coumarate:coenzyme A ligase, 4CL, Sugar release

## Abstract

**Background:**

The development of genome editing technologies offers new prospects in improving bioenergy crops like switchgrass (*Panicum virgatum*). Switchgrass is an outcrossing species with an allotetraploid genome (2*n* = 4*x* = 36), a complexity which forms an impediment to generating homozygous knock-out plants. Lignin, a major component of the plant cell wall and a contributor to cellulosic feedstock’s recalcitrance to decomposition, stands as a barrier to efficient biofuel production by limiting enzyme access to cell wall polymers during the fermentation process.

**Results:**

We developed a CRISPR/Cas9 genome editing system in switchgrass to target a key enzyme involved in the early steps of monolignol biosynthesis, 4-Coumarate:coenzyme A ligase (4CL). Three *4CL* genes, *Pv4CL1*, *Pv4CL2,* and *Pv4CL3*, were identified in switchgrass. Expression analysis revealed that *Pv4CL1* transcripts were more abundant in the stem than in the leaf, while *Pv4CL2* transcripts were barely detectable and *Pv4CL3* was mainly expressed in the leaf. *Pv4CL1* was selected as the target for CRISPR/Cas9 editing because of its preferential expression in highly lignified stem tissues. Specific guide RNA was constructed to target *Pv4CL1*. After introducing the construct into switchgrass calli, 39 transgenic plants were regenerated. Using two rounds of PCR screening and sequencing, four plants were confirmed to have tetra-allelic mutations simultaneously. The *Pv4CL1* knock-out plants had reduced cell wall thickness, an 8–30% reduction in total lignin content, a 7–11% increase in glucose release, and a 23–32% increase in xylose release.

**Conclusion:**

This study established a successful CRISPR/Cas9 system in switchgrass with mutation efficiency reaching 10%. The system allows the precise targeting of the selected *Pv4CL1* gene to create switchgrass knock-out mutant plants with decreased lignin content and reduced recalcitrance.

**Electronic supplementary material:**

The online version of this article (10.1186/s13068-017-0972-0) contains supplementary material, which is available to authorized users.

## Background

The production of biofuels from renewable biomass alleviates the dependence on fossil fuels, which has led to a strong interest in developing biofuel feedstock crops and new biofuel conversion technologies [1]. Switchgrass (*Panicum virgatum*), an outcrossing C4 perennial grass species, is a lignocellulosic feedstock for bioenergy. Lowland switchgrass (2*n* = 4*x* = 36) is a homoeologous allotetraploid with three genome classes of AA, AB, and BB [2]; homoeologous chromosomes result from a combinational pairing among four disomic inherited allelic chromosomes during cross-pollination [3]. The complex genome and outcrossing nature of switchgrass causes difficulty in producing homologous knock-out plants by genetic transformation or by TILLING.

In recent years, clustered regularly interspaced short palindromic repeats (CRISPR)/CRISPR-associated protein 9 (Cas9) technology (CRISPR/Cas9) has been developed that uses targeted nucleases to generate DNA breaks resulting in defined genetic modifications similar to, but much more exact than the modifications seen with conventional antisense, RNAi, and artificial microRNA techniques [4, 5]. The critical difference between the downregulation and CRISPR/Cas9 technologies is that the site-specific nucleases produce defined gene knock-outs by altering the genomic DNA sequence, while antisense, RNAi, and artificial microRNA techniques generate knock-downs by repressing transcription of the targeted gene [5]. The site-specific nuclease generates double-stranded breaks (DSBs), which are then repaired by either non-homologous end joining (NHEJ) or homologous recombination (HR). NHEJ is a common DSB repair mechanism in most organisms, including higher plants. NHEJ causes insertions and deletions (indels) of nucleotides (nt), which result in frameshift mutations causing disturbed translation of a coding gene [6–8].

The complex and costly deregulation process for conventional GMOs has made it extremely difficult to commercialize new transgenic cultivars [9, 10]. The situation is even more complicated in outcrossing perennial bioenergy species like switchgrass [10, 11]. However, plants obtained by genome editing technologies are considered low risk because these materials contain small genetic differences that can also occur naturally or through long-standing breeding methods. The US Department of Agriculture has determined that it will not regulate a waxy corn and a non-browning mushroom produced by CRISPR/Cas9 [12]. Therefore, the development of genome editing technologies offers new prospects in improving and commercializing switchgrass cultivars.

Genetic modification of lignin biosynthesis in switchgrass by RNAi has led to increased sugar release and improved bioethanol production [13–18]. Here, we employed the CRISPR/Cas9 system to generate low-lignin switchgrass with the intention to test the system in an outcrossing species with complicated genomes by producing total knock-outs of a gene previously only knocked down by RNAi. The CRISPR/Cas9 system has a relatively simple cloning method, which requires only a 20-bp sequence, upstream of the protospacer-adjacent motif (PAM), conferring sequence specificity for guide RNA designation. The target we selected for genetic modification is 4-coumarate:coenzyme A ligase (4CL), a key enzyme related to the early steps of the monolignol biosynthesis pathway [15]. 4CL catalyzes a conversion from *para*-coumaric acid to *para*-coumaroyl-CoA, which acts as a substrate for entry into the different branches of the pathway of phenylpropanoid metabolism [19]. Two switchgrass *4CL* genes (*Pv4CL*), *Pv4CL1* and *Pv4CL2*, had been previously identified in switchgrass [15]. In this study, we identified another *4CL* in switchgrass (*Pv4CL3*). Because *Pv4CL1* is highly expressed in stem tissues, we designed a CRISPR vector to specifically knock-out *Pv4CL1* in switchgrass. The efficiency of creating *pv4cl1* mutants by CRISPR reached 10% in this outcrossing tetraploid species. The mutant switchgrass plants showed reduced lignin and increase sugar release.

## Results

### *Pv4CL1* is preferentially expressed in the internode among three *4CLs* in switchgrass

**Fig. 1:**
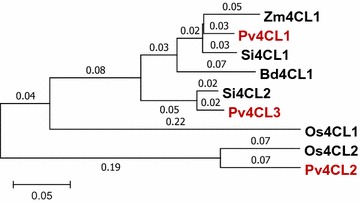
Phylogenic tree of Pv4CLs and their homologous genes in model plants. The scale indicates amino acid substitutions per position. Switchgrass, *Pv4CL1* (Pavir.Fa01395), *Pv4CL2* (Pavir.J20148), *Pv4CL3* (Pavir.Da00296); Brachypodium, Bd4CL1(Bradi3g05750.1); foxtail millet, Si4CL2(Si006172m), Si4CL1(Si016817m); rice, Os4CL2 (Q42982), Os4CL1 (BAD5189); maize, Zm4CL1 (AY566301). Red letters, switchgrass 4CLs

The precise genomic sequence of *Pv4CL* was required in order to find suitable target sites for guide RNA construction for the suppression of either *Pv4CL1* or *Pv4CL2*, or both *Pv4CL1* and *Pv4CL2. Pv4CL1* (EU491511.1) and *Pv4CL2* (JF414903) coding sequences were released on NCBI in 2011. Based on the two Pv4CLs and the Zm4CL (AY566301) coding sequences, we searched for the 4CL genomic sequences in a switchgrass genome database, switchgrass *v1*.*1* (Phytozome 2012; https://phytozome.jgi.doe.gov/pz/portal.html#). Blasting *Zm4CL* against *Panicum virgatum v1*.*1*, we discovered four coding sequences: Pavir.Fa01395, Pavir.J20148, Pavir.Da00296, and Pavir.Db00533. In a comparison between the four coding sequences and the two previously identified *Pv4CL*, identical matches were found—Pavir.Fa01395 to *Pv4CL1* (EU491511.1) and Pavir.J20148 to *Pv4CL2* (JF414903). Neither Pavir.Da00296 nor Pavir.Db00533 matched *Pv4CL1* and *Pv4CL2* completely; yet, Pavir.Da00296 and Pavir.Db00533 protein sequences contained all the characteristics of 4CL enzymes—the AMP-binding domain (187th to 199th and 188th to 200th), *GEICIRGR* motif (386th to 392nd and 387th to 393rd), the *VPP* (281st to 283rd and 282nd to 284th), and the *PVL* domains (340th to 342nd and 341st to 343rd) (Additional file 1: Figure S1) [20, 21]. Therefore, Pavir.Da00296 was renamed *Pv4CL3a* and Pavir.Db00533 was renamed *Pv4CL3b*. From a switchgrass genome annotation, the chromosomes of the genes were located: *Pv4CL1* on chromosome 6a, *Pv4CL2* on chromosome 1a, *Pv4CL3a* on chromosome 4a, and *Pv4CL3b* on chromosomes 4b. Between *Pv4CL3a* and *Pv4CL3b*, 97% protein identity was ascertained. Considering their locations and protein identities, *Pv4CL3a* and *Pv4CL3b* were possible alleles found in disomic inherited tetraploid plants, rather than two different genes. A phylogenetic tree with the three Pv4CLs and other 4CL proteins was drawn. Pv4CL1 was distant from Pv4CL2 and was in close proximity to Pv4CL3 (Fig. 1). Pv4CL1 had a 60% protein identity with Pv4CL2, and Pv4CL3 had an 83% and a 62% match to Pv4CL1 and Pv4CL2 protein sequences, respectively (Additional file 1: Figure S2).

**Fig. 2:**
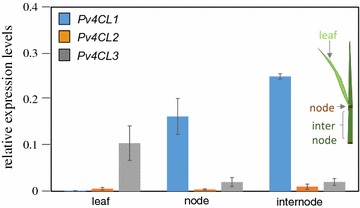
Quantitative RT-PCR analysis for expression levels of *Pv4CL1*, *Pv4CL2*, and *Pv4CL3* in switchgrass. *PvUbi* was used as a reference. Three tissues—leaf, node, internode—were sampled at the R1 developmental stage. Values represent mean ± SD of three biological replicates

Stems consist of internodes and nodes, which are highly lignified tissues composed of parenchyma and sclerenchyma cells distributed in the interfascicular region and the vascular sheath. To determine the major lignin targeting gene among *Pv4CL1*, *Pv4CL2* and *Pv4CL3*, their expression patterns were analyzed by qRT-PCR. Three tissues, internode, node, and leaf, were used for the analysis. *Pv4CL1* transcripts were more abundant in the internode and the node rather than in the leaf (Fig. 2, blue bars). *Pv4CL2* transcripts were barely detectable in the three different tissues (Fig. 2, orange bars), and *Pv4CL3* was preferentially expressed in the leaf only (Fig. 2, gray bars).

### *Pv4CL1* was targeted for lignin modification

Switchgrass genotype AP13 has been sequenced and the sequence information was released in Phytozyme. The sequences of *Pv4CLs* from AP13 were employed as references to identify corresponding genes in the tissue culture responsive genotype NFCX1. The AP13 genomic *Pv4CL1* sequence was amplified by Pv4CL1F12/Pv4CL1R12 primer pairs to single out the target region for genome editing in NFCX1. Flanking the target region of *Pv4CL1*, four single-nucleotide polymorphisms (SNPs) were discovered to distinguish subgenomes, A from B (Additional file 1: Figure S3). A 20-bp target site was designed before the CGG PAM in order to edit *Pv4CL1* (Additional file 1: Figure S3). The target site was compared to *Pv4CL2* and *Pv4CL3* genomic sequences from the switchgrass genotype NFCX1 (Additional file 1: Figure S3). The target site matched 17 nt/20 nt compared to *Pv4CL2*, and 19 nt/20 nt compared to *Pv4CL3* sequences (Additional file 1: Figure S3). The target site was cloned into the middle of OsU3:gRNA in a pRGEB32 vector [22], which carried Cas9. The resulting vector was transformed into the switchgrass genotype NFCX1 and 39 independent transgenic plants were obtained.

### Generation of homozygous mutants

In order to identify the edited *Pv4CL1* sequence in transgenic switchgrass plants, a serial PCR/sequencing method was designed (Additional file 1: Figure S4). Genomic DNA extracted from transgenic plants was used for PCR amplification with *Pv4CL1*-, *Pv4CL2*-, and *Pv4CL3*-specific primer pairs. The following PCR products were sequenced directly and were compared to the target region of the genotype NFCX1. Such target region comparisons led to the identification of *pv4cl* mutant plants: 25, 26, 28, and 29. While *Pv4CL2* and *Pv4CL3* had no edited mutants, the four plants (*Pv4cl*s-25, -26, -28, and -29) contained edited sequences in the target region of *Pv4CL1*. To further confirm the mutations, these four plants were used for a second round of PCR amplification with *Pv4CL1*-specific primers. The PCR products were cloned into a TA cloning vector. Twenty colonies for each individual edited plant were sequenced (Additional file 1: Figure S4).

**Fig. 3:**
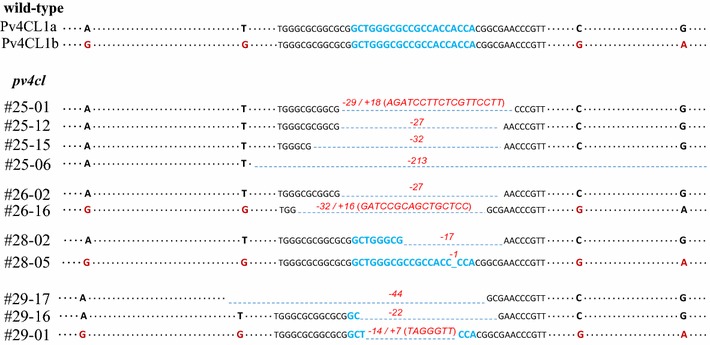
Results of 2nd PCR/sequencing showing mutations in *Pv4CL1*. *pv4cl1*-25 had four different mutation patterns, − 29/+ 18, − 27, − 32, and − 213; *pv4cl1*-26 had two different mutation patterns, − 27 and − 32/+ 16; *pv4cl1*-28 had two different mutation patterns, − 27 and − 1; *pv4cl1*-29 had three different mutation patterns, − 44, − 22, and − 14/+ 7 in *Pv4CL1*. Blue letters: the target site of *Pv4CL1*; dark red letters: SNPs; bright red letters: insertion sequences

According to the second round of sequencing, the four plants each had their own mutation patterns: a nucleotide deletion is indicated by a minus sign “−”, while an insertion is indicated by a plus sign “+” (Fig. 3). Four mutation patterns (− 29/+ 18; − 27; − 32; − 213) from the plant *pv4cl1*-25, two mutation patterns (− 27; − 32/+ 16) from *pv4cl1*-26, two mutation patterns (− 27; − 1) from *pv4cl*1-28, and three mutation patterns (− 44; − 22; − 14/+ 7) from *pv4cl1*-29 were identified (Fig. 3; Additional file 1: Figure S5). Amplicon deep sequencing of the lines confirmed *Pv4CL1* mutations in the alleles of subgenomes A and B, while no mutation was found in *Pv4CL2*, *Pv4CL3a*, and *Pv4CL3b*. The mutant plants were transferred to soil and grown in the greenhouse.

### Edited *pv4cl1* plants showed reduced lignin content

**Fig. 4:**
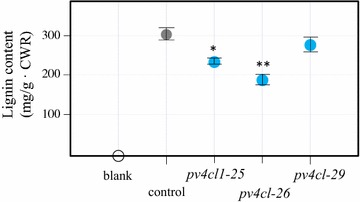
Total lignin content obtained by AcBr method. CWR: cell wall residue. Values represent mean ± SD of three biological replicates. *P < 0.05, **P < 0.01

Internodes and nodes excluding the leaf tissue were collected for analysis of lignin content and lignin-monomer composition. The R1 stage internodes of the *pv4cl1*-25, -26, -29 and the control were sampled to extract cell wall residue (CWR). The CWR was used for analysis of total lignin content by the acetyl bromide (AcBr) method and monomer composition by thioacidolysis. Plants *pv4cl1*-25, *pv4cl1*-26, and *pv4cl1*-29 showed 20, 30, and 8% reduction in AcBr lignin content compared to the control (Fig. 4). Thioacidolysis was carried out to analyze lignin monomers. Compared to control, both *pv4cl1*-25 and *pv4cl1*-26 exhibited 10 and 25% reduction of S lignin, and 45 and 51% reduction of G lignin, respectively, while no change of H lignin was observed in either. Similarly, but to a lesser degree, *pv4cl1*-29 showed an 8% reduction in S and G lignins and no change in H lignin. The S/G ratios were 1.2, 1.4, and 0.9 in *pv4cl1*-25, *pv4cl1*-26, and *pv4cl1*-29, respectively, while the S/G ratio of the control was 0.9 (Table 1).

**Table 1:** Monolignol compositions of control and mutant lines obtained by thioacidolysis assay (mg/g CWR)

	H	G	S	Total lignin	S/G ratio
Control 1	10 ± 1	140 ± 2	125 ± 2	275 ± 3	0.9
Control 2	10 ± 1	161 ± 3	143 ± 1	313 ± 3	0.9
*pv4cl1*-*25*	9 ± 1	92 ± 1	111 ± 2	213 ± 2	1.2
*pv4cl1*-*26*	9 ± 1	68 ± 1	95 ± 2	171 ± 1	1.4
*pv4cl1*-*29*	9 ± 1	131 ± 2	112 ± 2	251 ± 2	0.9

**Fig. 5:**
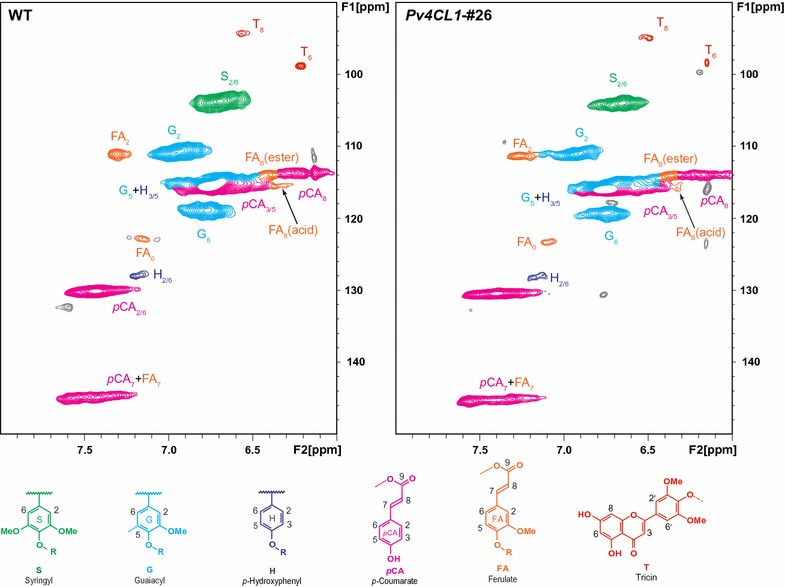
Aromatic regions of NMR spectra for the control and the gene-edited line *pv4cl1*-*26*

NMR analysis provided structural information of lignins including abundance of hydroxycinnamates and relative distribution of interunit linkages (Fig. 5; Additional file 2: Table S1). The content of hydroxycinnamates (*p*CA and FA) showed various levels of changes. Compared to control, ferulate (FA) in *pv4cl1*-25, -26, and -29 increased 2, 5, and 2%, respectively. For *p*-coumarate (*p*CA), the increases were 1, 26, and 10% (Additional file 2: Table S1). The composition of interunit linkages, *β*-*O*-4 (C–O bond), *β*-5 (C–C bond), and *β*–*β* (C–C bond), was analyzed regarding lignin polymer structure. The *β*-*O*-4 (C–O bond) was slightly increased in *pv4cl1*-25 and *pv4cl1*-26 plants and was not changed in *pv4cl1*-29. The *β*-5 (C–C bond) was slightly decreased in *pv4cl1*-25 and *pv4cl1*-26 plants but was not changed in *pv4cl1*-29. The *β*–*β* (C–C bond) was barely detectable in control and the mutant plants (Additional file 2: Table S1). Thus, the mutants did not show significant change in lignin polymer structure.

### *pv4cl1* mutants released more glucose/xylose than the control

The polysaccharide compositions of the cell wall were measured in the mutants and the control. The contents of glucose and xylose were slightly increased in *pv4cl1*-25 and *pv4cl1*-26, while *pv4cl1*-29 showed no change. The contents of arabinose and galactose were not changed in the *pv4cl1* plants (Additional file 2: Table S2).

**Fig. 6:**
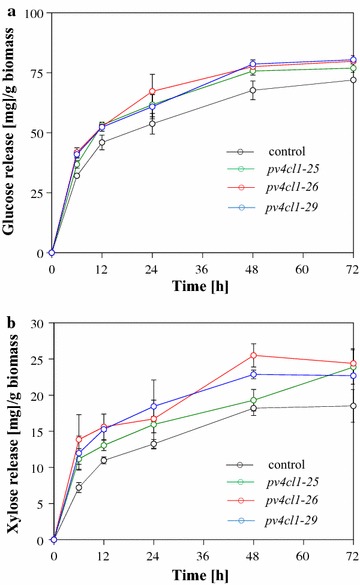
Glucose (**a**) and xylose (**b**) release in the *pv4cl*s mutants and the control. Values represent mean ± SD of three biological replicates

Biomass saccharification efficiency was analyzed by enzymatic hydrolysis without acid pretreatment. Enzymatic sugar (glucose/xylose) release was observed for 72 h with 6-, 12-, 24-, and 48-h intervals. Compared to control, more glucose and xylose were released in the mutants at different timepoints (Fig. 6). At 72 h, the *pv4cl1* mutant lines produced more glucose in the range of 7–11% and more xylose from 23 to 32% (Fig. 6).

### *pv4cl1* mutants had less lignin density and purple stem

**Fig. 7:**
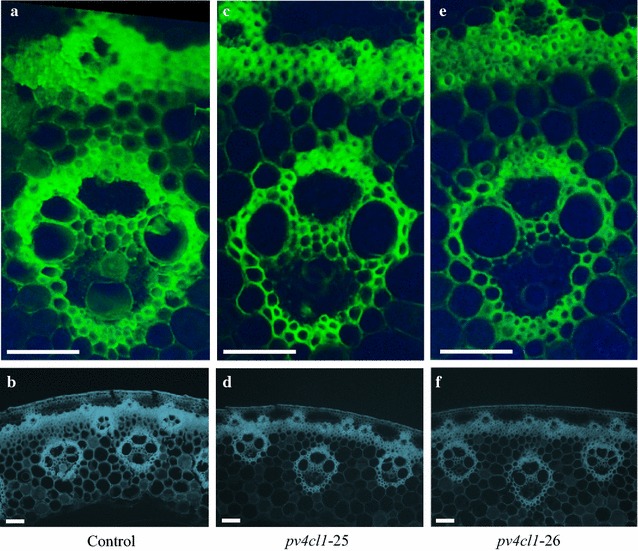
Histological staining for lignin deposition in control **a**, **b**, *pv4cl1*-25 **c**, **d** and *pv4cl1*-26 **e**, **f**. Second internode was sampled at R1 stage. Scale bars: 50 µm. Thickness of the cross section is 30 µm

Lignin distribution patterns in *pv4cl1*-25, -26 and control were observed through a histochemical assay, which contained 0.5% safranin-*O* and 0.5% toluidine blue-*O*. The two staining dyes showed that the primary cell wall embodied thin and bright green lines (Additional file 1: Figure S6), and the secondary cell wall had thick and dark green bands (Additional file 1: Figure S6) in a cross section of a switchgrass internode. Secondary cell wall was observed in the outer parenchyma cells and was mainly distributed in the interfascicular region and the vascular sheath, which were composed of sclerenchyma cells (Fig. 7; Additional file 1: Figure S6). Compared to the control, *pv4cl1*-25 and -26 showed thinner secondary cell wall in the sclerenchyma and in the parenchyma cells (Fig. 7c–f).

**Fig. 8:**
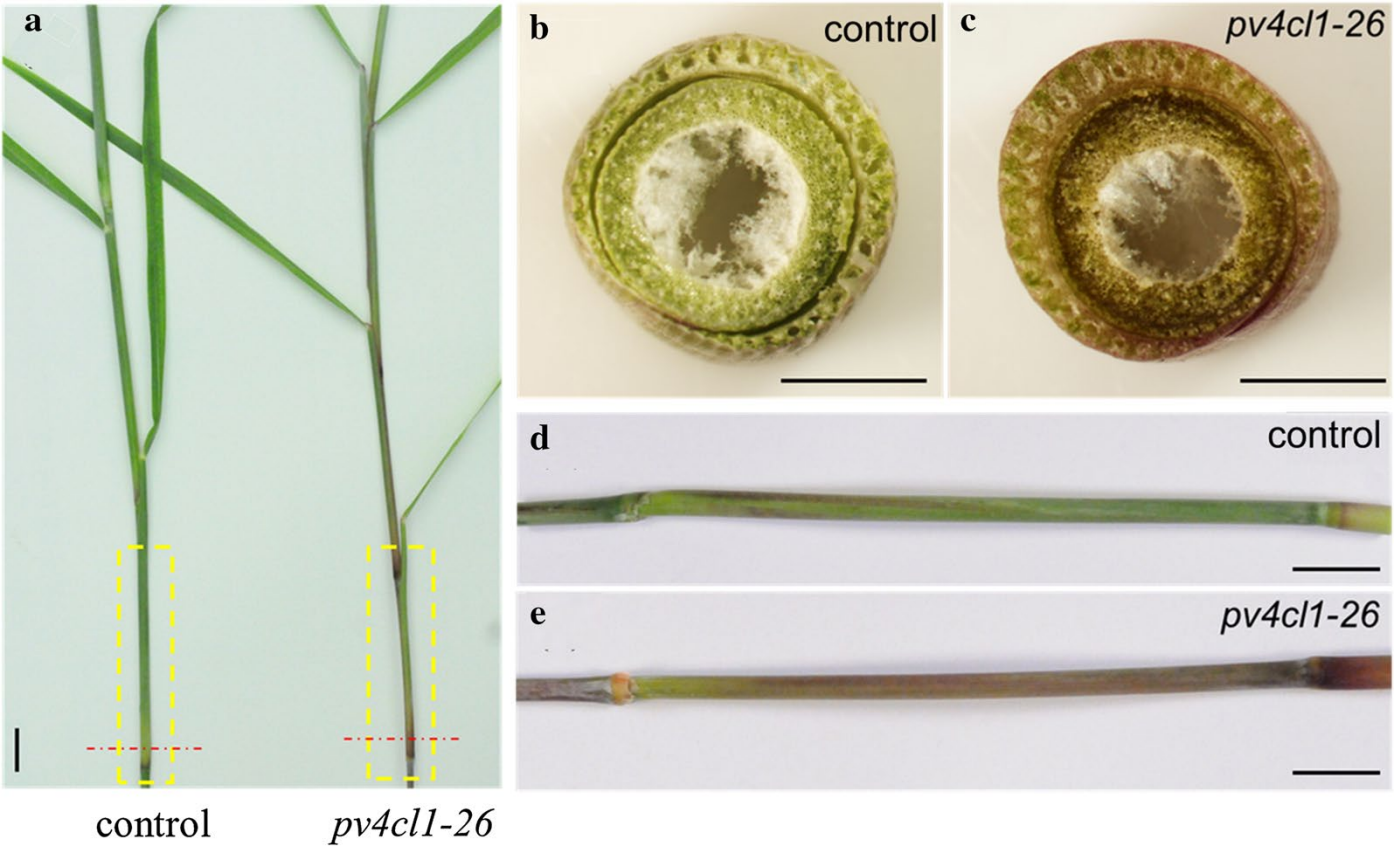
Comparison of stem color in control and a gene-edited line pv4cl1-26. **a** Tillers at reproductive stage. **b**, **c** Hand-sectioned stem without staining. **d**, **e** Magnified yellow rectangular boxes from **a**. Scale bar: 0.5 cm

At the seedling and vegetative stages, the *pv4cl1*-*26* plant showed normal green color in the leaf and the stem. However, at reproductive stage, the plant exhibited purple color in the stem (without staining), while the leaf color had no change (Fig. 8a–e).

## Discussion

### *Pv4CL3* was identified as a new member of the *Pv4CL* family and *Pv4CL1* was chosen as the target gene

Two homologous *4CL* genes, *Pv4CL1* and *Pv4CL2*, were previously identified in switchgrass [15]. Since the switchgrass genome sequence was updated in 2016 by Phytozyme (https://phytozome.jgi.doe.gov/pz/portal.html#), we searched again for homologous *4CL* genes and found *Pv4CL3* along with the previously identified *Pv4CL1* and *Pv4CL2*. According to a switchgrass genome annotation, *Pv4CL1*, *Pv4CL2*, and *Pv4CL3* are located on different chromosomes. Initial speculation was that the three 4CLs would display similar 4CL efficiencies if they showed similar protein identities. With 60% protein identity, Pv4CL1 and Pv4CL2 showed similar 4CL enzymatic efficiency [15]. With higher identities between Pv4CL3 and Pv4CL1 (83%) than to Pv4CL2 (62%), it is expected that Pv4CL3 may have similar enzyme activity similar to Pv4CL1.

The transcriptional expression of *Pv4CL1* was preferentially detected in highly lignified internodes rather than in leaf tissue. *Pv4CL2* was barely detected in leaves, nodes, and internodes. *Pv4CL3* was highly expressed in leaves, which were less lignified than nodes and internodes. Therefore, *Pv4CL1* was chosen as the target gene out of the three *Pv4CLs*.

### Homologous mutation in four *Pv4CL1* alleles is confounded by complex genetics

The mutation frequency of *Pv4CL1* by CRISPR/Cas9 in switchgrass reached 10% (4/39) after *Agrobacterium*-mediated transformation. We verified the 10% mutation rate using our serial PCR/sequencing method to demonstrate the specific changes in the sequences. It is known that mutation rate can be influenced by variations in the target sequences, the gRNA structure/sequence, the version of Cas9, and the gRNA expression strategy. Furthermore, quantification of mutation rate depends on the sensitivity of the analytical methods including PCR/T7 endonuclease I, PCR/restriction analysis, and PCR/direct sequencing [23]. Perhaps even more striking than optimizing these critical factors is the effect of increasing ploidy level and genome complexity on the efficiency of a CRISPR/Cas9 approach. It has been observed that the CRISPR/Cas9 mutation efficiency varied in the range of 1–84% of stable transformants in diploid Arabidopsis and rice [24], while the mutation frequency as detected through PCR/sequencing in allohexaploid winter wheat and spring wheat was 6–3%, respectively [23]. Even though considerations for the different factors of genome editing are important, we assert that complex-genome species naturally will have much lower mutation efficiency than simple genome species.

*Pv4CL1* has four alleles from two subgenomes (A and B); the flanking sequences of the target site in *Pv4CL1* have four single-nucleotide polymorphisms (SNPs) to distinguish A from B subgenomes. We obtained four plants with targeted mutations in which multiple types of deletions occurred at a single target site. The genome-edited plants each had their own mutation patterns. Acquiring four different mutation patterns is a reasonable outcome, as switchgrass has the ability to experience one to four independent editing events on four alleles. As an outcrossing species, it is important to have all four alleles simultaneously knocked out; this will significantly facilitate the breeding process.

### Mutant switchgrass plants had less lignin and higher sugar release

It was reported that RNAi lines of *Pv4CL1* resulted in a reduction of total lignin from 17 to 32% with a specific decrease in G lignin composition [15]. Our *pv4cl* mutants had 8–30% reduction in total lignin. RNAi lines of *Pv4CL1*, without acid pretreatment, obtained no differences in glucan and xylan yields [15]. However, our mutant plants, without acid pretreatment, produced from 7 to 11% higher glucose and 23–32% more xylose than the control. The sugar-release results indicate that the lignocellulosic bioethanol potential of knock-out plants, which contained indel mutations in *Pv4CL1*, was greater than by RNAi knock-down.

In summary, we developed a successful genome editing system in switchgrass, an outcrossing tetraploid bioenergy crop. We obtained tetra-allelic mutations in which all four alleles were simultaneously knocked out, a result that is significant for outcrossing or vegetatively propagated polyploid species. More importantly, by specifically targeting a 4CL homolog which has a preferential expression in highly lignified tissues, we produced mutant switchgrass plants that show reduced lignin content and improved sugar release. The ability to target and mutate one of the homologous genes is a unique advantage of genome editing that is unlikely to be achieved by RNAi technology.

## Methods

### Plant materials

Switchgrass genotype NFCX1 was used for genetic transformation and lignin modification. Switchgrass plants were grown in the greenhouse at 26 °C with 16 h light (390 µE m^−2^ s^−1^).

### Vector construction and plant transformation

Guide RNA was designed using CHOPCHOP v2 (chopchop.cbu.uib.no/), E-CRISP Design (http://www.e-crisp.org/), and the gRNA sequences were double-checked by a local blasting function provided by Bioedit (https://www.bioedit.com/). We downloaded switchgrass transcripts library from Phytozyme (https://phytozome.jgi.doe.gov/) and NCBI (https://www.ncbi.nlm.nih.gov/), and saved in Bioedit. We then screened several candidates and singled out the best spacer among the candidates through the local blast function of Bioedit. The spacers were cloned into pRGEB32, which contains the rice OsUbi2 promoter in front of Cas9 and the OsU3 promoter in front of gRNA [22]. The pRGEB32 carrying a 4CL spacer was transferred into the *Agrobacterium tumefaciens* strain AGL1. Transgenic switchgrass plants were obtained by *Agrobacterium*-mediated transformation following a previously described protocol [25]. All the transgenic switchgrass plants were regenerated from independent callus lines.

### Quantification of expression levels of endogenous genes by qRT-PCR

Leaves, nodes, and internodes of switchgrass plants at the R1 reproductive stage, according to Hardin et al. [26, 27], were sampled for a total RNA extraction. Total RNA was extracted using 1 ml TRI Reagent^®^ (Sigma, MO, USA) and 0.1 ml of 1-bromo-3-chloropropane (MRC, OH, USA) according to the manufacturer’s instructions. Sample mixtures were placed for 5 min at room temperature after shaking well. The sample mixtures were centrifuged for 15 min at 4 °C. After transferring 400 µl of supernatant, 300 µl isopropanol was added into a new 1.7-ml tube. The 1.7-ml tube containing supernatant was centrifuged for 8 min at 4 °C. The resulting pellet was washed with 75% EtOH. Two micrograms of RNA were reverse transcribed through reverse transcriptase using the Superscript III Kit (Invitrogen) and 18 mer oligo dT after treatment with TURBO™ DNase I (Ambion, Austin, TX). The primers were designed to amplify the 3´UTR sequences of *Pv4CL1*, *Pv4CL2*, and *Pv4CL3*, which are listed in Additional file 2: Table S3. *PvUbi* was used as a Ref. [28–31]. The Ct values of qRT-PCR were generated by ABI PRISM 7900 HT sequence detection system (Applied Biosystems). Changes in gene expression were calculated via the ΔΔ_Ct_ method.

### Quantification of lignin content and lignin-monomer composition

The internodes of transgenic plants at the R1 stage were harvested by removing the leaf blades and sheath. The internodes were chopped into 2–3 cm chips and then ground. The ground samples were washed three times by chloroform/methanol (2:1, v/v), methanol, and water as described by Chen et al. [26, 32]. The remaining cell wall residue (CWR) was lyophilized. The extractive-free CWR was used for quantifying lignin content and lignin-monomer compositions. The acetyl bromide (AcBr) method was used to quantify lignin content [33]. A 20 mg CWR was treated with 5 ml of 25% (*v*/*v*) acetyl bromide in glacial acetic acid for 4 h at 50 °C. The samples were then cooled and centrifuged at 3500 rpm for 5 min. Then, 4 ml of the top layer was transferred to a 50-ml volumetric flask, containing 10 ml of 2 M NaOH and 12 ml of acetic acid. One ml of 0.5 M hydroxylamine was added to each flask, and the samples were diluted to 50 ml with acetic acid. Absorption spectra (250–350 nm) were determined for each sample and were used to determine the absorption maxima at 280 nm. A molar extinction coefficient of 17.2 was used for all samples [32].

Lignin composition was determined using thioacidolysis method [34]. A 20 mg sample of CWR was treated with 3 ml of 0.2 M boron trifluoride etherate in an 8.75:1 dioxane/ethanethiol mixture for 4 h at 100 °C. After cooling, deionized water and saturated sodium bicarbonate were added, and the organic solvent was extracted twice with methane chloride and dried with anhydrous sodium sulfate. The solvent was dried under N_2_ gas and derivatized by adding 75 μl of pyridine and 75 μl MSTFA at 37 °C for 30 min. The derivatized samples were analyzed by GC/MS to measure the monolignol composition. Lignin-derived monomers (S, G, and H) were identified and quantified by Hewlett-Packard 5890 series II gas chromatograph with a 5971 series mass selective detector (column, HP-1, 60 m  ×  0.25 mm  ×  0.25 μm film thickness). Mass spectra were recorded in electron impact mode (70 eV) with 60–650 m/z scanning range [13].

### Lignin characterization using 2D HSQC NMR

Before conducting NMR characterization with switchgrass samples, each sample was soxhlet-extracted with DI water (24 h) and ethanol/toluene mixture (1:2, v/v) (12 h). The extractive-free samples were air-dried in a hood. About 500 mg of the extractive-free samples were ball-milled, and then hydrolyzed using enzyme mixtures, which included C-Tec2 and H-Tec2 in 20 mM sodium acetate buffer solution (pH 5.0) at 50 °C for 48 h (24 h × 2). The recovered lignin-enriched residues were treated with protease for 24 h to remove residual enzymes. Cellulolytic enzyme lignin (CEL) was extracted from the enzymatic residues by 96% toluene for 48 h. The extracts were concentrated via roto-evaporation and freeze-dried. The obtained lignin sample (~ 15 mg) was dissolved in 0.1 mL of DMSO-*d*_6_ for NMR analysis using a Shigemi NMR tube. NMR spectra were acquired at 298 K using a Bruker Avance III 400 MHz console equipped with a 5-mm BBO probe. Two-dimensional ^1^H–^13^C heteronuclear single quantum coherence (HSQC) spectra were collected using a Bruker standard pulse sequence (‘hsqcetgpsi2’). The central DMSO solvent peaks (*δ*_H_/*δ*_C_ = 2.49/39.5 ppm) were used for chemical shift calibration. Volume integration of cross peaks in HSQC spectra was carried out using Bruker’s TopSpin 2.1 software.

### Determination of sugar release

Dried, Wiley-milled switchgrass was analyzed for sugar-release efficiency. About 250 mg of samples (oven-dry weight) was loaded in 50 mM citrate buffer solution (pH 4.8) with Novozymes CTec2 (70 mg protein per g-biomass). Sugar release was conducted at 50 °C with 200 rpm in the incubator shaker. Liquid hydrolysate was periodically collected at 0, 6, 12, 24, 48, and 72 h, and enzymes in the hydrolysate were deactivated in the boiling water before carbohydrates analysis. Released sugars in each hydrolysate were measured using Dionex ICS-3000 ion chromatography system.

### Histochemical staining

Switchgrass internode samples from R1 stage tillers were collected in the greenhouse and immediately frozen in liquid nitrogen. The middle portions of internode two were cut into 30-μm sections with a Leica CM 1850 cryostat (Leica Microsystems Inc., Buffalo Grove, IL) at − 25 °C [26]. The cryosections were transferred to glass slides, thawed, stained, and covered with coverslips. Lignin was stained with 0.5% aqueous safranin-*O* (Sigma, St. Louis, MO) (*w*/*v*) dissolved in 50% EtOH or 0.5% aqueous toluidine blue-*O* (Sigma, St. Louis, MO) (*w*/*v*) dissolved in 50% EtOH. These stains increased the contrast of cell walls for bright field microscopy and at the same time reduced their autofluorescence when viewed under UV illumination [35]. Photographs were taken using a Nikon Optiphot-2 microscope system with NIS-Elements F3.0 (Nikon Instruments Inc., Laguna Hills, CA).

### Statistical analysis and phylogenetic analysis

Triplicate samples were collected from each edited line and the control. Data obtained from all biochemical or molecular analyses were subjected to one-way ANOVA. The difference between the edited lines and the control was evaluated by Student’s T test. Standard deviations (SD) are provided in all tables and figures as appropriate.

Phylogenetic analysis was done using Neighbor Joining of MEGA7 (http://www.megasoftware.net/). The phylogenic tree was linearized assuming equal evolutionary rates in all lineages. The evolutionary distances were computed using the Poisson correction method, and are in the units of the number of amino acid substitutions per site. Switchgrass, *Brachypodium*, and foxtail millet protein sequences were obtained from Phytozyme v.11, and rice and maize protein sequences were obtained from NCBI. Switchgrass, Pv4CL1 (Pavir.Fa01395), Pv4CL2 (Pavir.J20148), Pv4CL3 (Pavir.Da00296); *Brachypodium*, Bd4CL1 (Bradi3g05750.1); foxtail millet, Si4CL2 (Si006172m), Si4CL1 (Si016817m); rice, Os4CL2 (Q42982), Os4CL1 (BAD5189); maize, Zm4CL1 (AY566301).

## Additional files

**Additional file 1: Figure S1.** Protein sequence alignment between Pv4CL3a (Pavir.Da00296) and Pv4CL3b (Pavir.Db00533). *PYSSGTTGMPKGV* AMP-binding motif, *GEICIRGR* motif, *VPP* and *VPL* motifs are in red boxes. **Figure S2.** Identities among Pv4CL1 and its homologous proteins. **Figure S3.** Target region in the coding sequence of *Pv4CL1s* by CRISPR/Cas9. (a) Blue letters: a target site; dark red letters: SNPs. (b) *Pv4CL1* (Pavir.Fa01395), *Pv4CL2* (Pavir.J20148), *Pv4CL3a* (Pavir.Da00296) and *Pv4CL3b* (Pavir.Db00533) coding sequences were from *Panicum virgatum v1*.*1.* Red box: target site of *Pv4CL1*. Blue box: protospacer-adjacent motif (PAM). **Figure S4.** Schematic diagram of PCR/sequencing for screening of edited *Pv4CLs*. Steps in clockwise direction: (1) prepare transgenic plants; (2) extract genomic DNA; (3) amplify the target region of *Pv4CL1*, *Pv4CL2* and *Pv4CL3*; (4) sequence the PCR products; (5) sort-out edited lines; (6) re-amplify the target region of the edited lines with fresh genomic DNA; (7) clone the PCR products into a TA vector; (8) re-sequence inserts in a TA vector; (9) analyze lignin phenotype. **Figure S5.** Results of 2nd PCR/sequencing. *pv4cl1*-25 had four different mutation patterns, − 29/+ 18, − 27, − 32 and − 213; *pv4cl1*-26 had two different mutation patterns, − 27 and − 32/+ 16; *pv4cl1*-28 had two different mutation patterns, − 27 and − 1; *pv4cl1*-29 had three different mutation patterns, − 44, − 22 and − 14/+ 7 in Pv4CL1. Red box: target site of *Pv4CL1*. Blue box: PAM. **Figure S6.** Histological staining for lignin deposition. C: cortex; CW: cell wall; Ph: phloem; X: xylem; VS: vascular sheath cell; XP: xylem parenchyma cell; XT: xylem tracheid; XV: xylem vessels. Scale bar: 50 µm.

**Additional file 2: Table S1.** Semi-quantitative analysis of hydroxycinnamates and inter-linkages in switchgrass. FA and *p*CA compositions are expressed as a fraction of total lignin subunits (S + G + H); FA: ferulate; *p*CA: *p*-coumarate; β-*O*-4: β-aryl ether; β-5: phenylcoumaran; β–β: resinol. **Table S2.** Carbohydrate contents in the control and the mutants. **Table S3.** A list of primers used in this study.
