# Supplementary material for: Defined tetra-allelic gene disruption of the 4-coumarate:coenzyme A ligase 1 (Pv4CL1) gene by CRISPR/Cas9 in switchgrass results in lignin reduction and improved sugar release
Source: Biotechnol Biofuels. 2017 Nov 30;10:284. doi: 10.1186/s13068-017-0972-0 (PMC5708096; doi:10.1186/s13068-017-0972-0)
Supplement: Supplementary file 1 — Additional file 1: Figure S1. Protein sequence alignment between Pv4CL3a (Pavir.Da00296) and Pv4CL3b (Pavir.Db00533). PYSSGTTGMPKGV AMP-binding motif, GEICIRGR motif, VPP and VPL motifs are in red boxes. Figure S2. Identities among Pv4CL1 and its homologous proteins. Figure S3. Target region in the coding sequence of Pv4CL1s by CRISPR/Cas9. (a) Blue letters: a target site; dark red letters: SNPs. (b) Pv4CL1 (Pavir.Fa01395), Pv4CL2 (Pavir.J20148), Pv4CL3a (Pavir.Da00296) and Pv4CL3b (Pavir.Db00533) coding sequences were from Panicum virgatum v1.1. Red box: target site of Pv4CL1. Blue box: protospacer-adjacent motif (PAM). Figure S4. Schematic diagram of PCR/sequencing for screening of edited Pv4CLs. Steps in clockwise direction: (1) prepare transgenic plants; (2) extract genomic DNA; (3) amplify the target region of Pv4CL1, Pv4CL2 and Pv4CL3; (4) sequence the PCR products; (5) sort-out edited lines; (6) re-amplify the target region of the edited lines with fresh genomic DNA; (7) clone the PCR products into a TA vector; (8) re-sequence inserts in a TA vector; (9) analyze lignin phenotype. Figure S5. Results of 2nd PCR/sequencing. pv4cl1-25 had four different mutation patterns, − 29/+ 18, − 27, − 32 and − 213; pv4cl1-26 had two different mutation patterns, − 27 and − 32/+ 16; pv4cl1-28 had two different mutation patterns, − 27 and − 1; pv4cl1-29 had three different mutation patterns, − 44, − 22 and − 14/+ 7 in Pv4CL1. Red box: target site of Pv4CL1. Blue box: PAM. Figure S6. Histological staining for lignin deposition. C: cortex; CW: cell wall; Ph: phloem; X: xylem; VS: vascular sheath cell; XP: xylem parenchyma cell; XT: xylem tracheid; XV: xylem vessels. Scale bar: 50 µm. [file 13068_2017_972_MOESM1_ESM.pptx]

## Slide 1
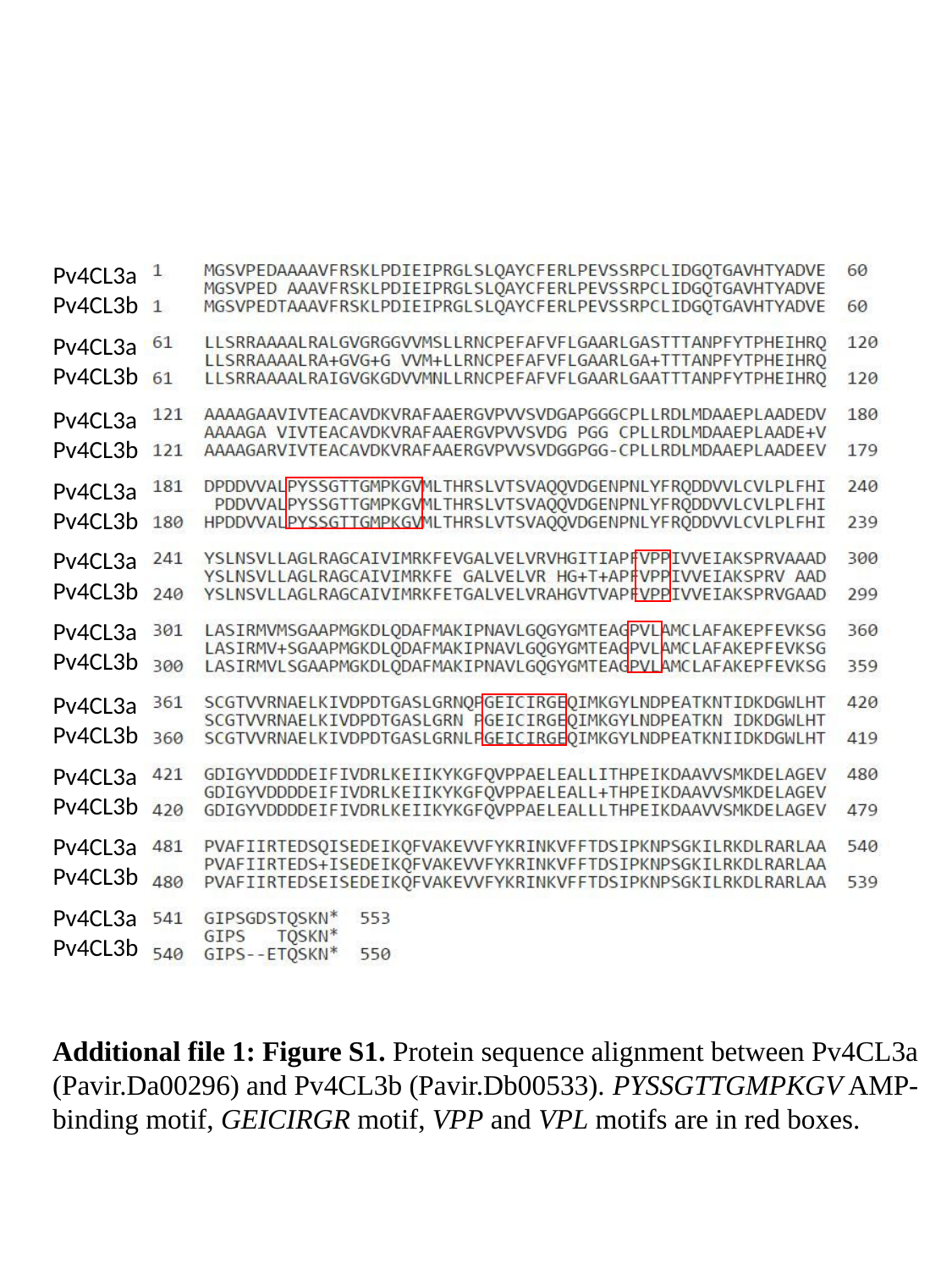

Pv4CL3a
Pv4CL3b
Pv4CL3a
Pv4CL3b
Pv4CL3a
Pv4CL3b
Pv4CL3a
Pv4CL3b
Pv4CL3a
Pv4CL3b
Pv4CL3a
Pv4CL3b
Pv4CL3a
Pv4CL3b
Pv4CL3a
Pv4CL3b
Pv4CL3a
Pv4CL3b
Pv4CL3a
Pv4CL3b
Additional file 1: Figure S1. Protein sequence alignment between Pv4CL3a (Pavir.Da00296) and Pv4CL3b (Pavir.Db00533). PYSSGTTGMPKGV AMP- binding motif, GEICIRGR motif, VPP and VPL motifs are in red boxes.

## Slide 2
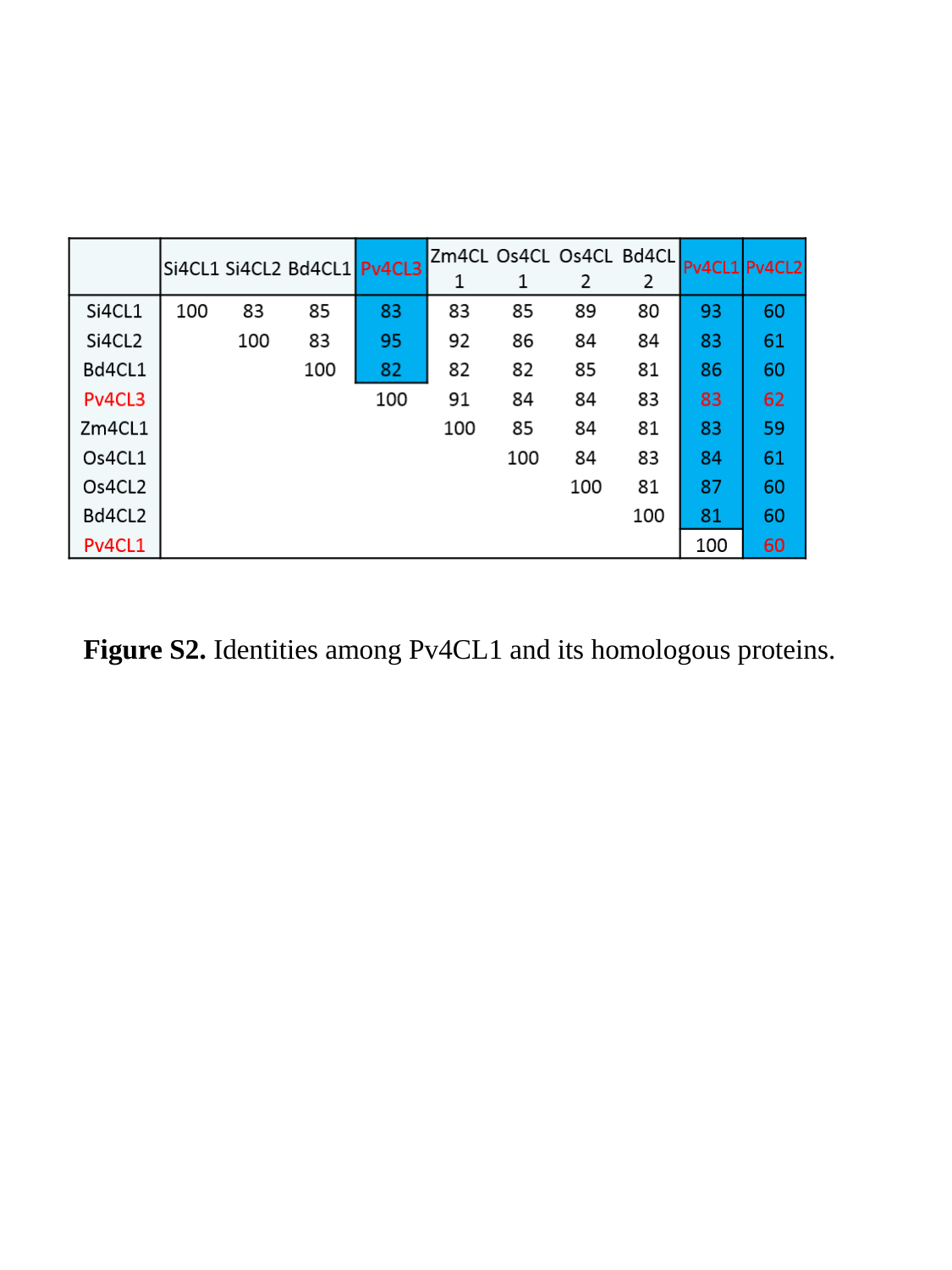

Figure S2. Identities among Pv4CL1 and its homologous proteins.

## Slide 3
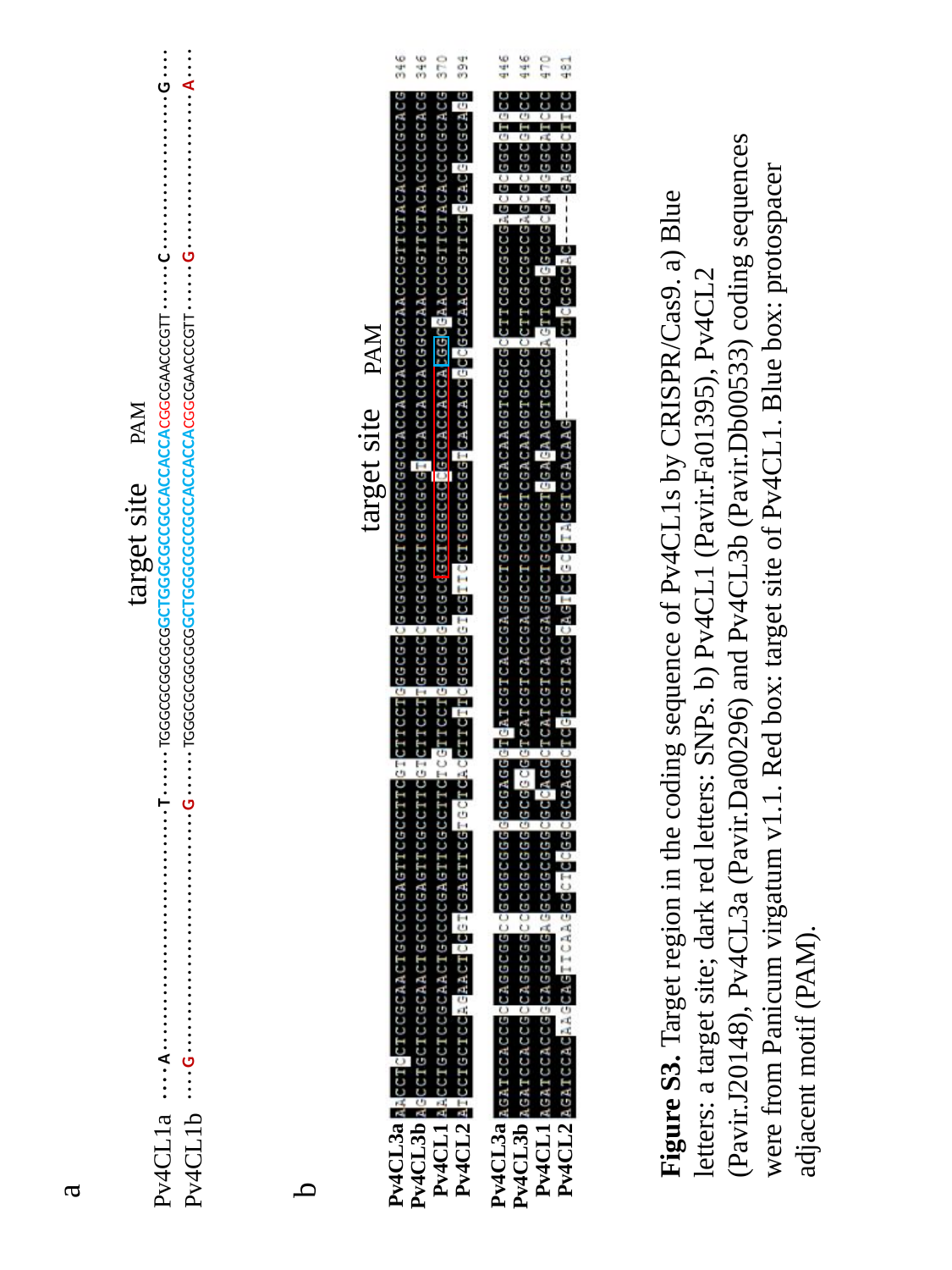

· · · · G · · · · · · · · · · · · · · · · · · · · · · · · · · · · · · · G · · · · · · TGGGCGCGGCGCGGCTGGGCGCCGCCACCACCACGGCGAACCCGTT · · · · · · G · · · · · · · · · · · · · · · · · · · · A · · · ·
 · · · · A · · · · · · · · · · · · · · · · · · · · · · · · · · · · · · · T · · · · · · TGGGCGCGGCGCGGCTGGGCGCCGCCACCACCACGGCGAACCCGTT · · · · · · C · · · · · · · · · · · · · · · · · · · · G · · · ·
 PAM
 target site
PAM
Pv4CL3a
Pv4CL3b
Pv4CL1
Pv4CL2
Pv4CL3a
Pv4CL3b
Pv4CL1
Pv4CL2
 target site
Figure S3. Target region in the coding sequence of Pv4CL1s by CRISPR/Cas9. a) Blue letters: a target site; dark red letters: SNPs. b) Pv4CL1 (Pavir.Fa01395), Pv4CL2 (Pavir.J20148), Pv4CL3a (Pavir.Da00296) and Pv4CL3b (Pavir.Db00533) coding sequences were from Panicum virgatum v1.1. Red box: target site of Pv4CL1. Blue box: protospacer adjacent motif (PAM).
Pv4CL1a
Pv4CL1b
a
b

## Slide 4
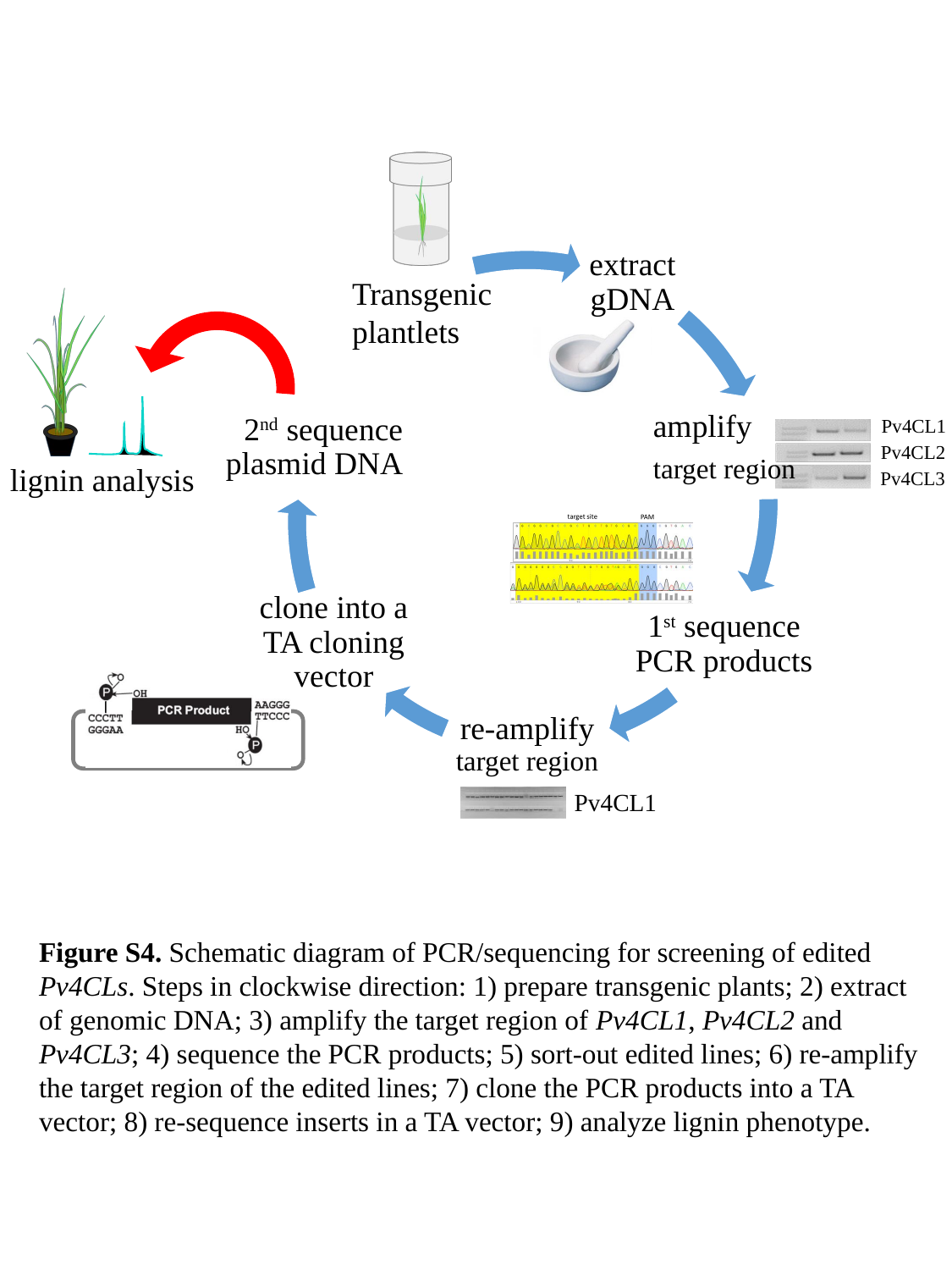

Transgenic plantlets
Pv4CL1
Pv4CL2
lignin analysis
Pv4CL3
Pv4CL1
Figure S4. Schematic diagram of PCR/sequencing for screening of edited Pv4CLs. Steps in clockwise direction: 1) prepare transgenic plants; 2) extract of genomic DNA; 3) amplify the target region of Pv4CL1, Pv4CL2 and Pv4CL3; 4) sequence the PCR products; 5) sort-out edited lines; 6) re-amplify the target region of the edited lines; 7) clone the PCR products into a TA vector; 8) re-sequence inserts in a TA vector; 9) analyze lignin phenotype.

## Slide 5
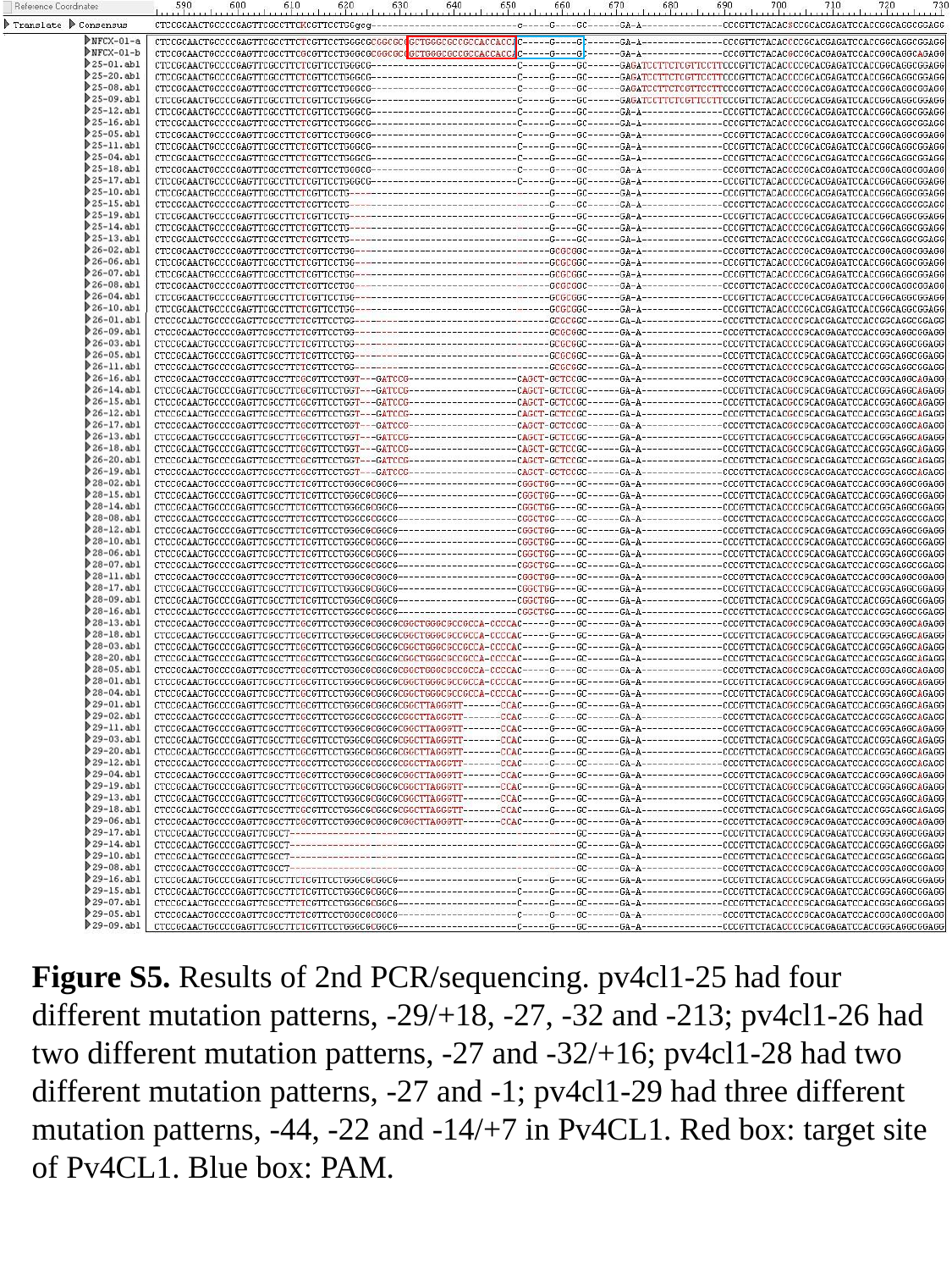

Figure S5. Results of 2nd PCR/sequencing. pv4cl1-25 had four different mutation patterns, -29/+18, -27, -32 and -213; pv4cl1-26 had two different mutation patterns, -27 and -32/+16; pv4cl1-28 had two different mutation patterns, -27 and -1; pv4cl1-29 had three different mutation patterns, -44, -22 and -14/+7 in Pv4CL1. Red box: target site of Pv4CL1. Blue box: PAM.

## Slide 6
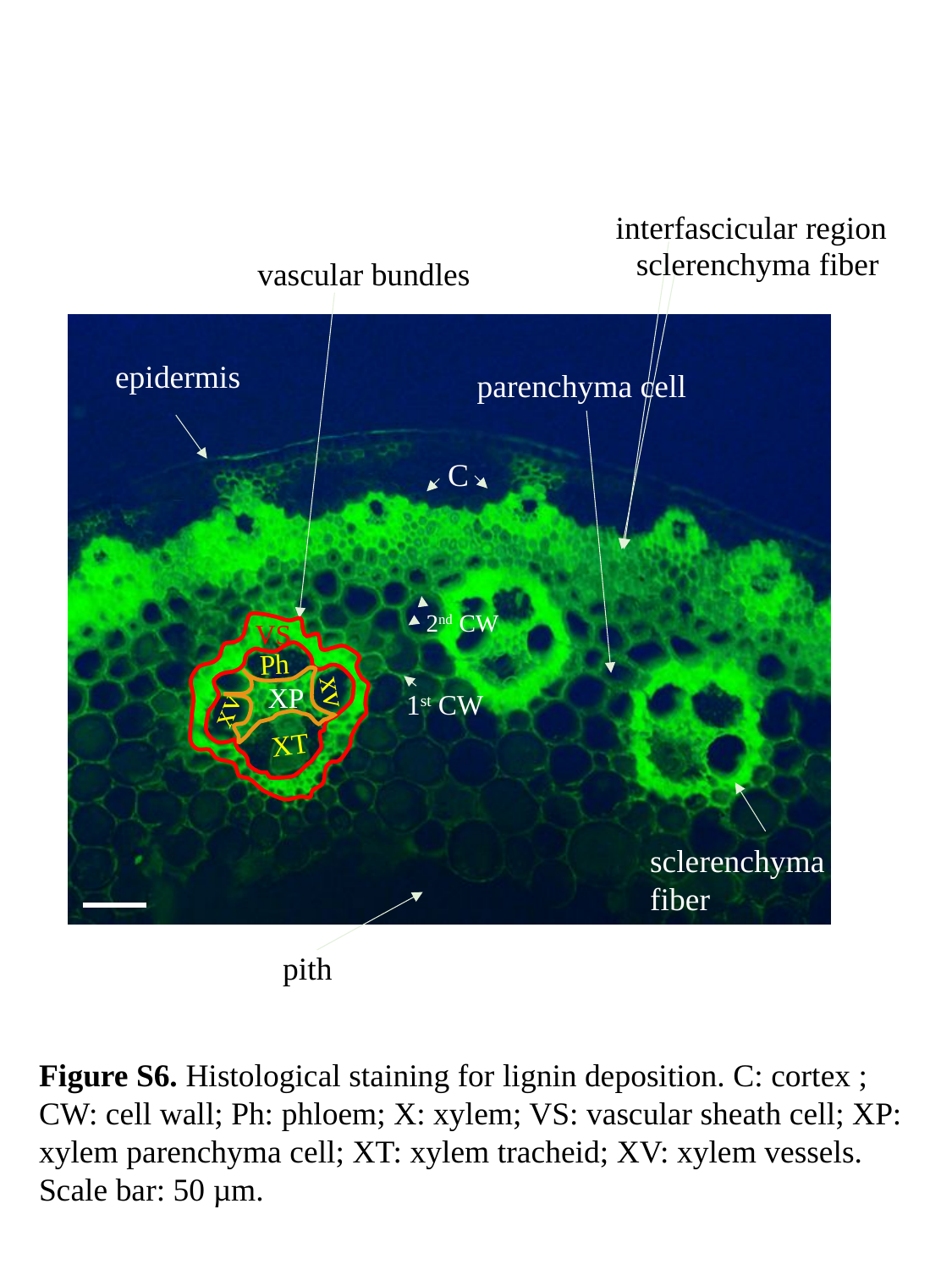

interfascicular region
sclerenchyma fiber
vascular bundles
epidermis
parenchyma cell
C
2nd CW
VS
Ph
XP
1st CW
xv
xv
XT
sclerenchyma fiber
pith
Figure S6. Histological staining for lignin deposition. C: cortex ; CW: cell wall; Ph: phloem; X: xylem; VS: vascular sheath cell; XP: xylem parenchyma cell; XT: xylem tracheid; XV: xylem vessels. Scale bar: 50 µm.
