# Supplementary material for: Defined tetra-allelic gene disruption of the 4-coumarate:coenzyme A ligase 1 (Pv4CL1) gene by CRISPR/Cas9 in switchgrass results in lignin reduction and improved sugar release
Source: Biotechnol Biofuels. 2017 Nov 30;10:284. doi: 10.1186/s13068-017-0972-0 (PMC5708096; doi:10.1186/s13068-017-0972-0)
Supplement: Supplementary file 2 — Additional file 2: Table S1. Semi-quantitative analysis of hydroxycinnamates and inter-linkages in switchgrass. FA and pCA compositions are expressed as a fraction of total lignin subunits (S + G + H); FA: ferulate; pCA: p-coumarate; β-O-4: β-aryl ether; β-5: phenylcoumaran; β–β: resinol. Table S2. Carbohydrate contents in the control and the mutants. Table S3. A list of primers used in this study. [file 13068_2017_972_MOESM2_ESM.pptx]

## Slide 1
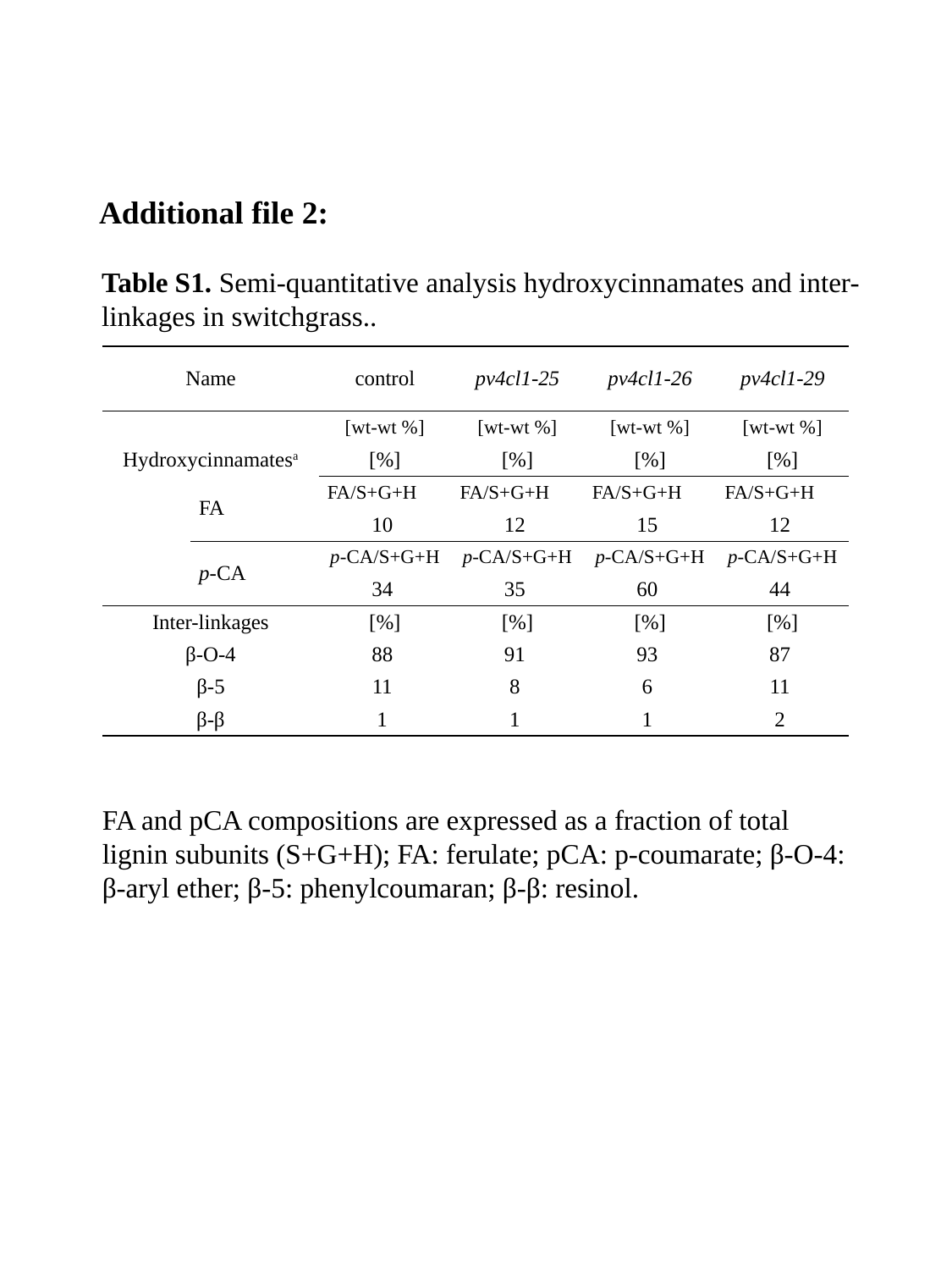

Additional file 2:
Table S1. Semi-quantitative analysis hydroxycinnamates and inter-linkages in switchgrass..
| Name | | control | pv4cl1-25 | pv4cl1-26 | pv4cl1-29 |
| --- | --- | --- | --- | --- | --- |
| | | [wt-wt %] | [wt-wt %] | [wt-wt %] | [wt-wt %] |
| Hydroxycinnamatesa | | [%] | [%] | [%] | [%] |
| | FA | FA/S+G+H | FA/S+G+H | FA/S+G+H | FA/S+G+H |
| | | 10 | 12 | 15 | 12 |
| | p-CA | p-CA/S+G+H | p-CA/S+G+H | p-CA/S+G+H | p-CA/S+G+H |
| | | 34 | 35 | 60 | 44 |
| Inter-linkages | | [%] | [%] | [%] | [%] |
| β-O-4 | | 88 | 91 | 93 | 87 |
| β-5 | | 11 | 8 | 6 | 11 |
| β-β | | 1 | 1 | 1 | 2 |
FA and pCA compositions are expressed as a fraction of total lignin subunits (S+G+H); FA: ferulate; pCA: p-coumarate; β-O-4: β-aryl ether; β-5: phenylcoumaran; β-β: resinol.

## Slide 2
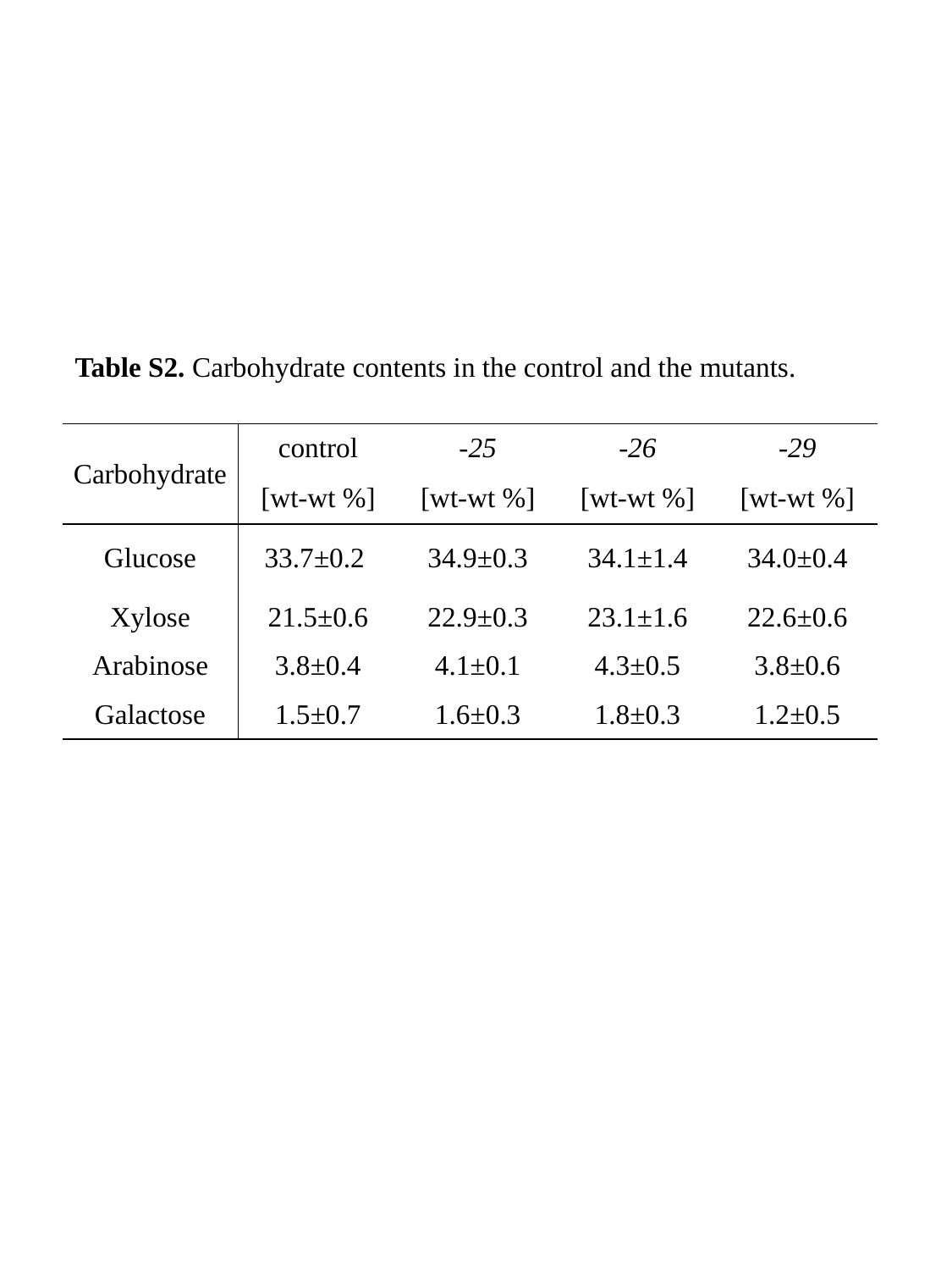

Table S2. Carbohydrate contents in the control and the mutants.
| Carbohydrate | control | -25 | -26 | -29 |
| --- | --- | --- | --- | --- |
| | [wt-wt %] | [wt-wt %] | [wt-wt %] | [wt-wt %] |
| Glucose | 33.7±0.2 | 34.9±0.3 | 34.1±1.4 | 34.0±0.4 |
| Xylose | 21.5±0.6 | 22.9±0.3 | 23.1±1.6 | 22.6±0.6 |
| Arabinose | 3.8±0.4 | 4.1±0.1 | 4.3±0.5 | 3.8±0.6 |
| Galactose | 1.5±0.7 | 1.6±0.3 | 1.8±0.3 | 1.2±0.5 |

## Slide 3
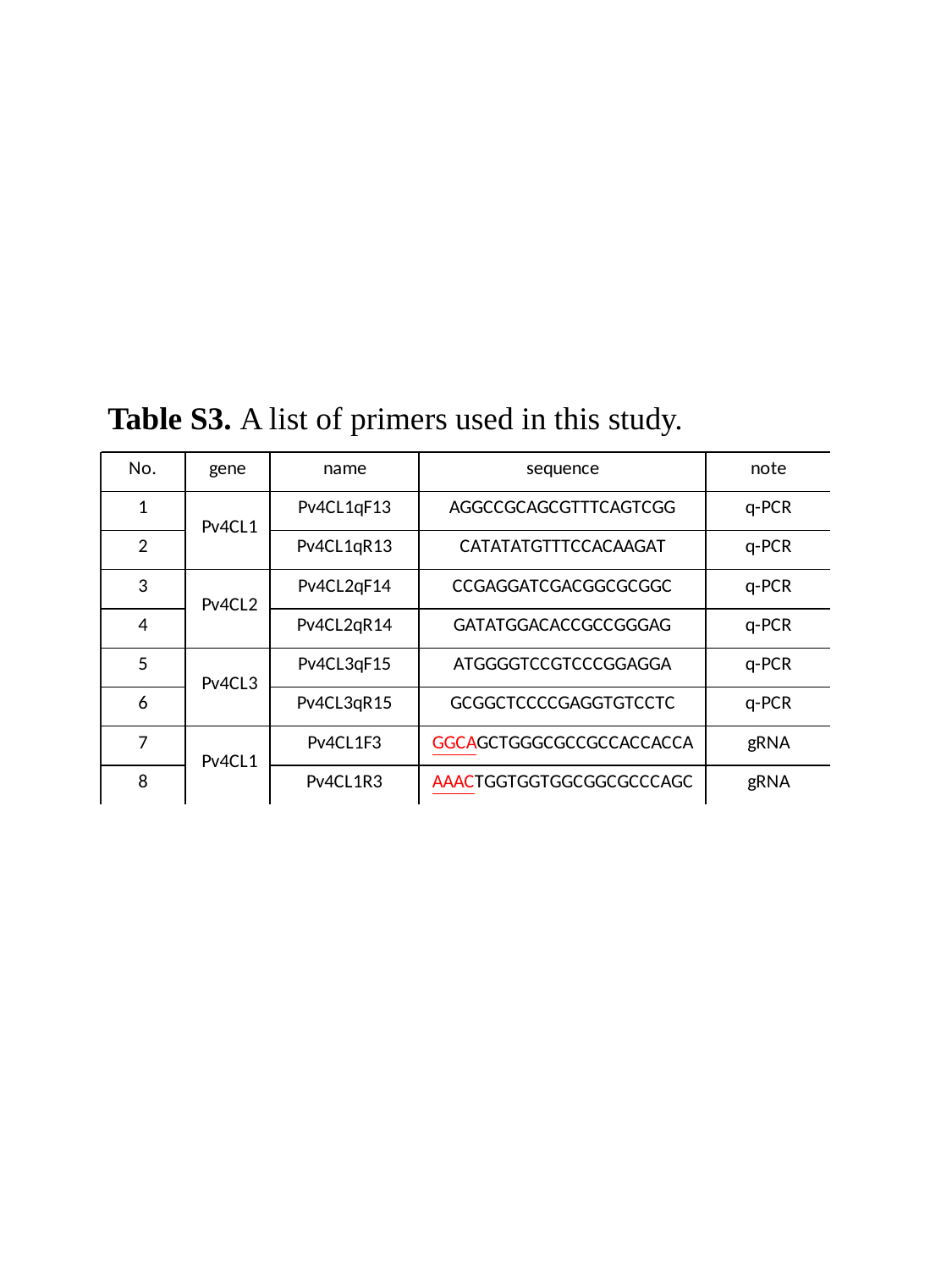

Table S3. A list of primers used in this study.
